# Dietary n-3 Fatty Acids Inhibit Fever Induced by Inflammation in the Rat

**DOI:** 10.1155/S0962935194000499

**Published:** 1994

**Authors:** A. L. Cooper, A. V. Turnbull, S. J. Hopkins, N. J. Rothwell

**Affiliations:** 1 Dept Physiological Sciences University of Manchester Oxford Road Manchester M13 9PT UK; 2 Rheumatic Diseases Centre Hope Hospital Salford M6 8HD UK

## Abstract

Modification of endogenous eicosanoid synthesis by dietary n-3 fatty acid supplementation reduces febrile responses, but the mechanisms underlying these effects *in vivo* have not been determined. In the present study, local inflammation was induced by intramuscular injection ofturpentine in rats fed control or n-3 supplemented diets for 8-9 weeks. In animals fed the control diet, turpentine induced fever, hypermetabolism, marked local inflammation (oedema), increased plasma IL-6 concentrations and raised cerebrospinal fluid (CSF) concentrations of PGE2. N-3 fatty acid supplementation significantly inhibited the rise in CSF PGE2, fever and hypermetaboHsm induced by turpentine. Local inflammation and increased plasma IL-6 concentrations were not affected by n-3 supplementation. These findings suggest that modification of dietary fat intake inhibits fever via reduced release of prostaglandins, probably within the brain, but does not affect the local or afferent signals involved in fever generation.


Research Paper

Mediators of Inflammation 3, 353–357 (1994)

### Dietary n-3 fatty acids inhibit fever induced by inflammation in the rat

A. L. Cooper,[1] A. V. Turnbull,[1] S. J. Hopkins[2] and N. J. Rothwell[1,CA]

[1]Dept Physiological Sciences, University of Manchester, Oxford Road, Manchester, M13 9PT, UK and [2]Rheumatic Diseases Centre, Hope Hospital, Salford M6 8HD, UK

[CA] Corresponding Author

**MODIFICATION of endogenous eicosanoid synthesis by dietary n-3 fatty acid supplementation reduces febrile responses, but the mechanisms underlying these effects *in vivo* have not been determined. In the present study, local inflammation was induced by intramuscular injection of turpentine in rats fed control or n-3 supplemented diets for 8–9 weeks. In animals fed the control diet, turpentine induced fever, hypermetabolism, marked local inflammation (oedema), increased plasma IL-6 concentrations and raised cerebrospinal fluid (CSF) concentrations of $PGE_2$. N-3 fatty acid supplementation significantly inhibited the rise in CSF $PGE_2$, fever and hypermetabolism induced by turpentine. Local inflammation and increased plasma IL-6 concentrations were not affected by n-3 supplementation. These findings suggest that modification of dietary fat intake inhibits fever via reduced release of prostaglandins, probably within the brain, but does not affect the local or afferent signals involved in fever generation.**

**Key words:** Diet, Fever, Hypermetabolism, IL-6, Local inflammation, n-3 fatty acids, Rat

### Introduction

Fever is one of the most common manifestations of disease, and is observed following injury, infection, malignant or inflammatory disease.[1] The release and action of several cytokines, including interleukin-1 (IL-1), IL-6 and tumour necrosis factor-α (TNFα), appear to play important roles in the development of fever. However, it is the concentration of IL-6 in plasma that correlates most closely with the rise in body temperature, making this cytokine the best candidate as a circulating endogenous pyrogen. IL-6 or other endogenous pyrogens are thought to act on or near thermoregulatory centres in the brain and increase body temperature via the release of prostaglandins which act directly on thermosensitive neurons within the hypothalamus (see Reference 2).

Eicosanoids (including prostaglandins and leukotrienes) directly mediate the pyrogenic action of the cytokines IL-1, IL-6 and TNFα but may also be involved in other pathways involved in the generation of a fever. For example, prostaglandins and leukotrienes are mediators of the local inflammatory reaction,[3] and can modify production of pyrogenic cytokines *in vitro*.[4,5]

Endogenous eicosanoid synthesis can be modified by dietary supplementation with n-3 fatty acids such as those present in fish oil. These n-3 fatty acids suppress normal, endogenous eicosanoid synthesis (PG of the 2 series and LT of the 4 series) and produce less reactive derivatives (PG of the 3 series and LT of the 5 series.[6–8] Several studies have investigated the effect of n-3 fatty acid supplementation on the pathways involved in fever generation. N-3 supplemented diets reduce fever and hypermetabolism induced by IL-1 in experimental animals,[9,10] or by typhoid vaccine in humans.[11] In addition, it has been shown that dietary n-3 fatty acids inhibit local inflammatory responses[8] and cytokine production *in vitro*.[11–13] To date, however, no studies have compared the effect of n-3 fatty acids on all of these pathways *in vivo*.

In the present study, we have investigated the effect of dietary n-3 supplementation on the local responses, afferent and central signals involved in fever generation. Fever and the associated hypermetabolism were induced by intramuscular administration of turpentine. We have previously shown that this protocol induces sustained increases in body temperature and thermogenesis which are associated with local inflammation and a marked rise in the plasma concentration of IL-6 but not IL-1 or TNF.[14,15] In addition, we have also demonstrated[14] that the fever and hypermetabolism induced by turpentine injection is dependent on cyclo-oxygenase products (most probably the PG) released within the central nervous system.

### Methods

Male, Sprague–Dawley rats (Charles River, Kent, UK), weighing approximately 40 g (21 days old) were caged in pairs at 24°C with a 12 h light/dark cycle (0800–2000). Animals were fed either a semisynthetic control or an n-3 supplemented diet (supplied by Dr

© 1994 Rapid Communications of Oxford Ltd

G. Livesey, AFRC Institute of Food Research, Norwich, UK) for 9 weeks. The diets had similar metabolizable energy density (control 15.7 kJ/g, n-3 16.3 kJ/g) and composition (22% protein) apart from their fatty acid content (Table 1). n-9 Fatty acids were the principal lipid source in the control diet while the experiment diet contained a micro-gelatin encapsulated fish-oil formula containing sucrose, ascorbate and tri-calcium phosphate (Dry n-3, Danochem, Ballerup, Denmark). Because the Dry n-3 formula comprised only 8.75% n-3 fatty acids, the final concentration of n-3 fatty acids in the experimental diet (per kg) was 1.75% (0.9% eicosapentaenoic acid, 0.6% docosahexaenoic acid and 0.25% of other n-3 fatty acids). In addition, a minimum amount (1%) of n-6 fatty acids was incorporated into the experimental diet to prevent essential fatty acid deficiency. After 8–9 weeks of supplementation, the livers of control and n-3 supplemented rats were removed for phospholipid analysis using high-pressure liquid chromatography (performed by Scotia Pharmaceuticals, Nova Scotia, Canada).

A sterile abscess was induced by intramuscular (i.m.) injection of 0.6 ml of turpentine (Rowney, Berkshire, UK) into conscious, hand-held rats.[14] An equal volume of sterile saline was injected i.m. into control animals. All measurements were made 18–20 h (0800–1000) after the i.m. injection.

Changes in metabolic rate and body temperature were determined by the measurement of oxygen consumption ($Vo_2$) and colonic temperature (Tc), respectively. $Vo_2$ was measured in closed-circuit indirect calorimeters[16] maintained at 24°C, for periods of 2–4 h. Animals were placed in individual calorimeters which allowed some movement (e.g. turning, stretching etc.). Resting values for $Vo_2$ were taken as the minimum values over 5 min periods (in order to minimize the contribution of physical activity), and are presented as the mean resting rate of $Vo_2$ per min, corrected for temperature, pressure and metabolic body size ($kg^{0.75}$). Stable, reproducible values were obtained over the duration of measurement (2 h), although the first 30 min period was excluded, to allow the animals to settle down in the calorimeters. Tc was determined immediately after measurements of $Vo_2$ by the insertion of a plastic-coated thermocouple connected to an electronic thermometer (Comark, Sussex, UK), 6 cm beyond the anus in hand-held rats.

After measurement of $Vo_2$ and Tc, some animals were decapitated and trunk blood was collected for subsequent determination of plasma IL-6 concentration using the B9 hybridoma bioassay.[21] Values are expressed as international units/ml (recDNA Human IL-6, 88/514, NIBSC, South Mimms).

After measurements of Tc and $Vo_2$, some animals were anaesthetized by the intravenous administration of sodium pentobarbitone (100 mg/kg) and cerebrospinal fluid (CSF) was collected by exposure and puncture of the cisterna magna. $PGE_2$ concentration was measured in the collected CSF by an in-house radioimmunoassay. Briefly, samples were incubated with $^3H$-$PGE_2$ and a $PGE_2$ antibody (donated by Dr F. Carey, Zeneca, Cheshire, UK) for 18 h. The unbound $^3H$-$PGE_2$ was removed by centrifugation after addition of a 1% charcoal, 2% dextran solution. The remaining supernatant was then counted using a β scintillation counter. The detection limit of the assay was 10 pg/ml.

Limb oedema (inflammation) was assessed from the percentage water content. Upon sacrifice, the injected limb was severed along the inguinal region and weighed immediately. The limb was then dried in air at 70°C for 7 days to constant weight. Thereafter the limb was reweighed (dry weight) and the percentage water content calculated, taking into account the injected volume (0.6 ml).

Values are presented as means ± standard errors ($n$ = 8–11 animals). Statistical analysis was performed using an unpaired Student's $t$-test or two-way ANOVA for two and four groups respectively. In all cases, two-tailed probabilities of less than 0.5 were considered statistically significant.

**Table 1.** Percentage composition of the control and n-3 supplemented diet

| Dietary constituent | Diet | |
|---|---|---|
| | Control | n-3 |
| Casein | 20 | 20 |
| Corn starch | 49 | 43 |
| DL-Methionine | 0.002 | 0.002 |
| Vitamin mix | 2 | 2 |
| Mineral mix | 4 | 4 |
| Sugar | 13 | 10 |
| Sunflower oil | 0.001 | 1 |
| Olive oil | 6.1 | – |
| **Dry n-3 formula** | – | 20 |
| n-3 Fatty acids | – | 8.75 |
| Gelatin | 4.4 | – |
| Ca ascorbate | 0.8 | – |
| Tri-calcium phosphate | 0.2 | – |
| Water | 0.5 | – |

### Results

Rats fed the n-3 supplemented diet weighed slightly more at the time of experimentation (472 ± 5 *vs.* controls 448 ± 10, $n$ = 20, $p < 0.05$ Student's $t$-test). This 5% difference in body weight probably reflects the slightly greater energy density of the n-3 supplemented diet as food intake was similar between the two groups (data not shown). The phospholipid composition of livers from n-3 supplemented rats was significantly different from that in control fed animals (Table 2). The largest changes were apparent in n-3 and n-6 fatty acid content, in that n-3 supplemented animals had significantly lower concentrations of n-6 tissue phospholipid and elevated concentrations of n-3 phospholipids than rats fed the control diet (Table 2).

n-3 Supplementation had no effect on the percentage water content of the limbs of animals injected with saline (control, 63.46 ± 0.67%, n-3, 61.55 ± 0.48%; Fig. 1). Injection of turpentine into animals fed the control diet caused a significant increase in the water content of the injected limb 18 h post-injection (76.34 ± 0.31%, $p < 0.001$, two-way ANOVA) and this response was not affected by n-3 supplementation (75.65 ± 0.615, Fig. 1).

Turpentine injection was associated with a marked elevation (1990 ± 380 IU/ml) in plasma IL-6 concentration measured 18–20 h post-injection (Fig. 2, $p < 0.001$, two-way ANOVA). This response was similar in turpentine injected animals receiving the n-3 supplemented diet.

The concentration of $PGE_2$ in the CSF of n-3 supplemented rats injected with saline was 48% lower than that observed in control fed rats injected with saline (45 ± 11 pg/ml) *vs.* 83 ± 27 pg, $p < 0.01$, Student's *t*-test, Fig. 3). Turpentine administration caused a 5.5-fold increase in CSF $PGF_2$ levels in animals fed the control diet (463 ± 25 pg/ml). The magnitude of this response was significantly reduced (by 75%) in n-3 supplemented rats injected with turpentine (177 ± 34 pg/ml, $p < 0.01$, two-way ANOVA).

Eighteen to 20 h after intramuscular injection of saline, values for $Vo_2$ were similar for control and n-3 supplemented animals (control 12.5 ± 1.1 ml/min/kg$^{0.75}$ *vs.* n-3 11.0 ± 0.4 ml/min/kg$^{0.75}$). Animals fed the control diet and injected with turpentine exhibited a significant hypermetabolism at this time

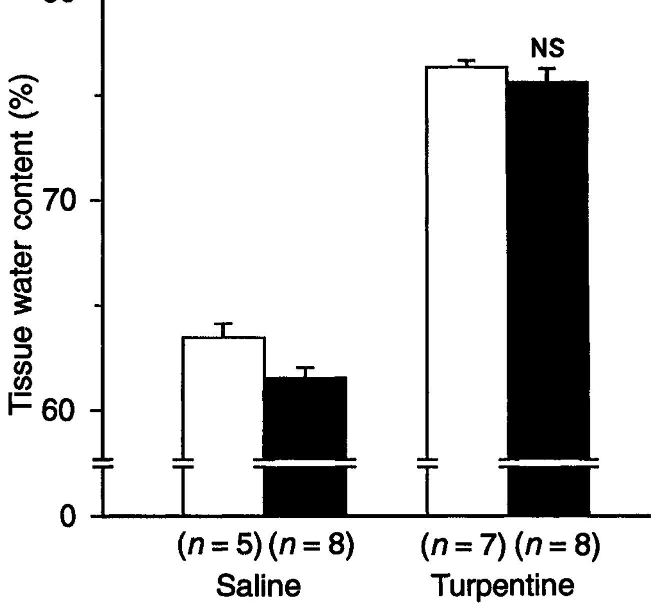

FIG. 1. Limb oedema assessed by percentage tissue water content in rats fed either a control or n-3 supplemented diet, 18 h after intramuscular injection of turpentine or saline. Mean ± S.E. $n = 5$–8. □, control; ■, n-3.

(17.2 ± 1.0 ml/min/kg$^{0.75}$), 37% above that of their respective saline group (Fig. 4). Feeding with the n-3 supplemented diet significantly reduced the hypermetabolic response to turpentine injection ($p < 0.05$, two-way ANOVA), although $Vo_2$ was still elevated by 28% above that of n-3 supplemented animals injected with saline.

Tc was similar for both saline-injected groups 20 h post-injection (control fed 36.4 ± 0.2°C, n-3 supplemented 36.2 ± 0.2°C, Fig. 4). In control animals injected with turpentine, a significant fever (Tc = 38.6 ± 0.2°C) was observed. The magnitude of this response was significantly reduced, although not abolished, in the n-3 supplemented animals (37.4 ± 0.2, $p < 0.05$ two-way ANOVA).

**Table 2.** Fatty acid composition of the livers of animals fed either the control or n-3 supplemented diet for 8–9 weeks. Mean ± S.E. Statistical analysis by unpaired Student's *t*-test

| Fatty acid | Control ($n = 12$) | n-3 ($n = 12$) | Change (%) | *p* value |
|---|---|---|---|---|
| 14.0 | 0.195 ± 0.014 | 0.252 ± 0.018 | 29 | < 0.05 |
| 16.0 | 16.612 ± 0.249 | 20.242 ± 0.309 | 22 | < 0.001 |
| 16.1n7 | 3.169 ± 0.235 | 2.724 ± 0.104 | –14 | NS |
| 18.0 | 14.426 ± 0.907 | 13.957 ± 0.790 | –3 | NS |
| 18.1n9 | 13.219 ± 0.321 | 7.730 ± 0.226 | –42 | < 0.001 |
| 18.2n6 | 9.298 ± 0.282 | 6.828 ± 0.252 | –36 | < 0.001 |
| 18.3n6 | 0.299 ± 0.029 | 0.078 ± 0.048 | –74 | < 0.001 |
| 18.3n3 | 0.034 ± 0.016 | 0.184 ± 0.012 | 44 | < 0.001 |
| 20.2n6 | 2.769 ± 0.121 | 0.244 ± 0.017 | –91 | < 0.001 |
| 20.3n6 | 2.255 ± 0.082 | 0.713 ± 0.016 | –68 | < 0.001 |
| 20.4n6 | 27.029 ± 1.119 | 10.00 ± 0.285 | –170 | < 0.001 |
| 20.5n3 | 0.363 ± 0.019 | 13.80 ± 0.573 | 3702 | < 0.001 |
| 22.4n6 | 0.371 ± 0.018 | 0.235 ± 0.025 | –37 | < 0.001 |
| 22.5n6 | 2.936 ± 0.155 | 0.215 ± 0.014 | –93 | < 0.001 |
| 22.5n3 | 0.392 ± 0.018 | 3.912 ± 0.164 | 898 | < 0.001 |
| 22.6n3 | 5.646 ± 0.244 | 17.200 ± 0.449 | 205 | < 0.001 |
| Others | 0.165 ± 0.031 | 0.181 ± 0.017 | –10 | NS |

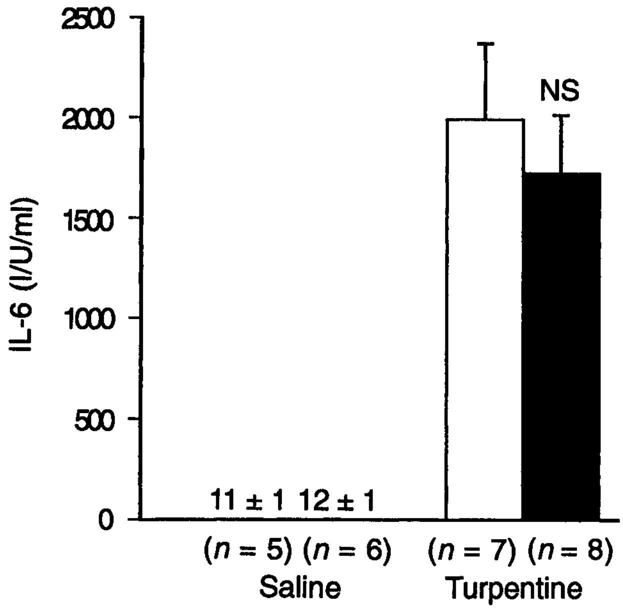

FIG. 2. Plasma interleukin-6 concentrations (IL-6, IU/ml) in rats fed either a control or n-3 supplemented diet, 18 h after intramuscular injection of turpentine or saline. Mean ± S.E. $n = 5$–8. □, control; ■, n-3.

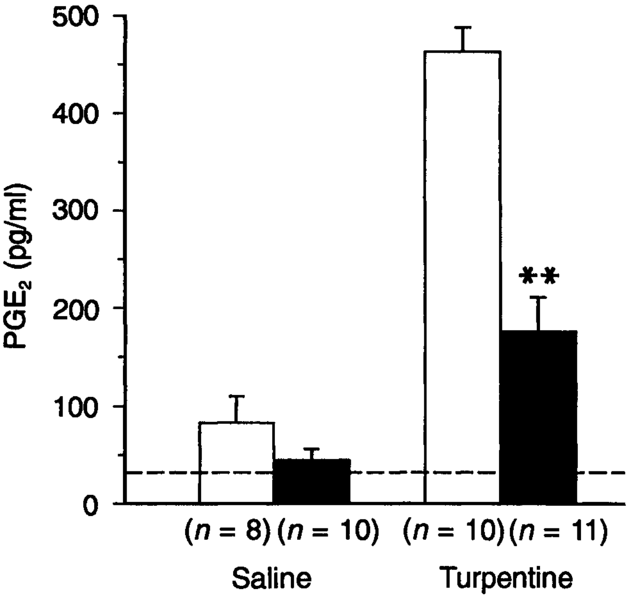

FIG. 3. Cerebrospinal fluid (CSF) concentrations of prostaglandin $E_2$ ($PGE_2$) in rats fed either a control or n-3 supplemented diet, 18 h after intramuscular injection of turpentine or saline. Mean ± S.E. $n = 8$–11. Detection limit of assay = 10 pg/ml. $^{**}p < 0.01$, two-way ANOVA *vs.* turpentine injection in animals fed the control diet. □, control; ■, n-3.

### Discussion

The results of the present study demonstrate that the pyrogenic and thermogenic responses to localized inflammation can be reduced by dietary n-3 fatty acid modification. n-3 Supplementation also attenuated the increases in the CSF concentrations of $PGE_2$ induced by turpentine, but did not affect the local inflammation or the plasma concentration of IL-6.

It is well established that n-3 fatty acid supplementation alters membrane composition,[17,18] and this was evident in the phospholipid composition of liver

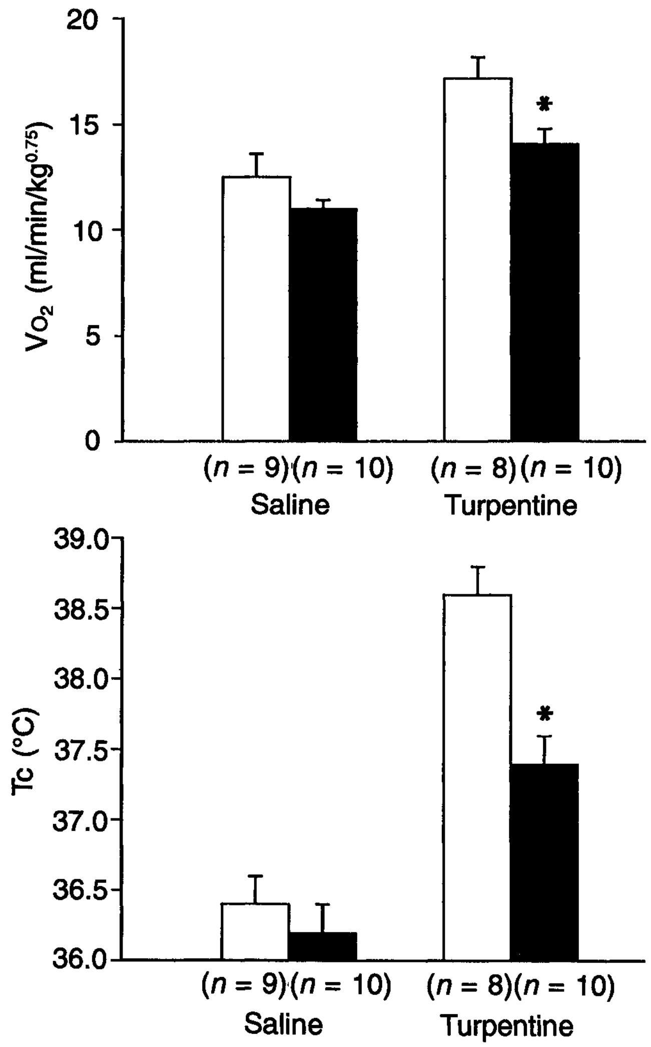

FIG. 4. Oxygen consumption ($Vo_2$, upper panel) and colonic temperature (Tc, lower panel) in rats fed either a control or n-3 supplemented diet, 18–20 h after intramuscular injection of turpentine or saline. Mean ± S.E. $n = 8$–10. $^{*}p < 0.05$ two-way ANOVA *vs.* turpentine injection in animals fed the control diet. □, control; ■, n-3.

membranes performed in the present study. Of particular relevance is the significant reduction in arachidonic acid (20:4n6) content, and the increase in membrane eicosapentaenoic acid (20:5n3) and docosahexaenoic acid (22:6n3) content in n-3 supplemented animals. The former is the common precursor for prostaglandin and leukotriene synthesis (of the 2 and 4 series respectively), whilst the latter compete with arachidonic acid as substrates for the cyclo-oxygenase and lipo-oxygenase enzymes, and produce less bioactive derivatives.[6–8]

Although membrane composition of brain tissue was not determined in the present study, the reduced production of $PGE_2$ in the CSF of turpentine injected animals suggests that the period of n-3 supplementation (9 weeks) was sufficient to alter membrane composition within the central nervous system. Central administration of cyclo-oxygenase inhibitors markedly attenuates the fever and hypermetabolism observed 18–20 h after turpentine injection.[14] Thus the responses observed in n-3 supplemented animals in the present study were probably due to reduced release of PG within the brain resulting from altered membrane phospholipid composition, although other effects of dietary fatty acid modification cannot be excluded.

Prostaglandins have been ascribed an integral role in fever, and are believed to act as the final common mediators of fever by altering the firing pattern of thermosensitive neurons and thus increasing the set-point for body temperature. Prostaglandin release within the brain mediates the fever induced by turpentine since this response is attenuated by administration of cyclo-oxygenase inhibitors directly into the brain,[14] and is associated with a marked increase in CSF $PGE_2$ concentration as demonstrated in the present study. Although n-3 fatty acids are known to alter eicosanoid synthesis, and have been shown to attenuate fever after IL-1 administration in experimental animals,[9,10] typhoid vaccine injection in human volunteers,[12] and turpentine injection in the rat (present study); the stage at which n-3 fatty acids act in the fever cascade *in vivo* was, however, unclear.

Although *in vitro* studies have shown that cytokine production can be modified by n-3 fatty acid supplementation,[13,19] *in vivo* circulating cytokine concentrations have not previously been studied after n-3 fatty acid supplementation. In the present study, plasma IL-6 concentrations were not affected by n-3 supplementation. IL-1 and TNFα are also involved in the fever and hypermetabolism observed after turpentine injection (Cooper *et al.*, unpublished data; Turnbull *et al.*, unpublished data), and contribute to the rise in plasma IL-6. Production of all three cytokines has been shown to be sensitive to n-3 fatty acid supplementation in *in vitro* endotoxin stimulation studies, though the effect has differed between species. In man, n-3 fatty acid supplementation inhibits IL-1 production and has little effect upon TNF production,[12,19] whereas, in mice both IL-1 and TNF production are increased.[13,20] However, it is difficult to compare these varied data for cytokine production *in vivo* and *in vitro* in response to different stimuli. The local inflammatory response to turpentine injection (water content of the injected limb) was similar in control and n-3 supplemented animals, indicating that dietary modification did not influence the extent of tissue damage. This contrasts with data showing effects of n-3 supplementation on inflammation in response to other stimuli and may reflect different mechanisms underlying tissue damage in these studies.[8] However, pharmacological inhibition of prostaglandin synthesis also has little effect on limb oedema after turpentine (Turnbull and Rothwell, unpublished).

In summary, n-3 fatty acid supplementation has been shown to attenuate the fever and hypermetabolism observed after turpentine injection. The authors suggest that this effect may be due to reduced production of $PGE_2$ within the central nervous system in n-3 fatty acid supplemented rats and not reduced inflammation *per se* or attenuated release of afferent cytokine signals.

### References

1. Atkins E, Bodel P. Clinical fever: its history, manifestations and pathogenesis. *Fed Proc* 1979; **38:** 57–63.
2. Kluger MJ. Fever: role of pyrogens and cryogens. *Physiol Rev* 1991; **71:** 93–127.
3. Salmon JA, Higgs GA. Prostaglandins and leukotrienes as inflammatory mediators. *Brit Med Bull* 1987; **43:** 285–296.
4. Kunkel SL, Chensue SW, Phan SH. Prostaglandins as endogenous mediators of interleukin-1 production. *J Immunol* 1986; **136:** 186–192.
5. Rola-Pleszczynski M, Lemaire I. Leukotrienes augment IL-1 production by human monocytes. *J Immunol* 1985; **135:** 3958–3961.
6. Needleman P, Raz A, Minkes MS, Ferrendelli JA, Spreche H. Triene prostaglandins, prostacyclin and thromboxane biosynthesis and unique biological properties. *Proc Natl Acad Sci* 1979; **76:** 944–948.
7. Lokesh BR, Black JM, German JB, Kinsella JE. Docosahexaenoic acid and other dietary polyunsaturated fatty acids suppress leukotriene synthesis by mouse peritoneal macrophages. *Lipids* 1988; **23:** 968–972.
8. Terano T, Salmon JA, Higgs GA, Moncada S. Eicosapentaenoic acid as a modulator of inflammation. Effect on prostaglandin and leukotriene synthesis. *Biochem Pharmacol* 1986; **35:** 779–785.
9. Pomposelli JJ, Mascioli Ea, Bistrian BR, Lopes SM, Blackburn GL. Attenuation of the febrile response in guinea-pigs by fish oil enriched diets. *J Parenteral and Enteral Nutrition* 1980; **13:** 136–140.
10. Cooper AL, Rothwell NJ. Inhibition of the thermogenic and pyrogenic responses to interleukin-1β in the rat by dietary n-3 fatty acid supplementation. *Prost Leuk & Ess Fatty Acids* 1993; **49:** 615–626.
11. Billiar TR, Bankey PE, Svingen BA, *et al.* Fatty acid intake and Kupffer cell function: fish oil alters eicosanoid and monokine production to endotoxin stimulation. *Surgery* 1988; **104:** 323–349.
12. Cooper AL, Gibbons L, Horan MA, Little RA, Rothwell NJ. Effect of dietary fish oil supplementation on fever and cytokine production in human volunteers. *Clin Nutr* (in press) 1993.
13. Hardardottir I, Kinsella JE. Tumor necrosis factor production by murine resident peritoneal macrophages is enhanced by dietary n-3 polyunsaturated fatty acids. *Biochim Biophys Acta* 1991; **1095:** 187–195.
14. Cooper AL, Rothwell, NJ. Mechanisms of the early and later hypermetabolism and fever after localized tissue injury in the rat. *Am J Physiol* 1991; **261:** E698–705.
15. Cooper AL, Kirkman E, Little RA, Hopkins SJ, Rothwell NJ. Mechanisms involved in the fever and hypermetabolism induced by turpentine injection in the rat. *Proc Nut Soc* 1991; **50:** 149A.
16. Stock MJ. An automated, closed circuit oxygen consumption apparatus for small animals. *J Appl Physiol* 1975; **39:** 849–850.
17. German JB, Lokesh B, Bruckner GG, Kinsella JE. Effect of increasing dietary fish oil on tissue lipids and prostaglandin synthesis in the rat. *Nutr Res* 1985; **5:** 1393–1407.
18. Philbrick DJ, Mahadevappa VG, Ackman RG, Holub BJ. Ingestion of fish oil or a derived n-3 fatty acid concentrate containing eicosapentaenoic acid (EPA) affects fatty acid compositions of individual phospholipids of rat brain, sciatic nerve and retina. *J Nutr* 1987; **117:** 1663–1670.
19. Endres S, Ghorbani R, Kelley VE, *et al.* The effect of dietary supplementation with n-3 polyunsaturated fatty acids on the synthesis of interleukin-1 and tumor necrosis factor by mononuclear cells. *New Eng J Med* 1989; **320:** 265–271.
20. Lokesh BR, Sayers TJ, Kinsella JE. Interleukin-1 and tumour necrosis factor synthesis by mouse peritoneal macrophages is enhanced by dietary n-3 polyunsaturated fatty acids. *Immunol Lett* 1990; **23:** 281–286.
21. Holt I, Cooper RA, Hopkins SJ. Relationship between local inflammation, interleukin-6 concentration and the acute phase protein response in arthritic patients. *Eur J Clin Invest* 1991; **21:** 479–484.

**Received 29 March 1994; accepted in revised form 25 May 1994**

## References

[PDF_00450] Atkins E., Bodel P. (1979). Clinical fever: its history, manifestations and pathogenesis.. Fed Proc.

[PDF_00474] Billiar T. R., Bankey P. E., Svingen B. A., Curran R. D., West M. A., Holman R. T., Simmons R. L., Cerra F. B. (1988). Fatty acid intake and Kupffer cell function: fish oil alters eicosanoid and monokine production to endotoxin stimulation.. Surgery.

[PDF_00471] Cooper A. L., Rothwell N. J. (1993). Inhibition of the thermogenic and pyrogenic responses to interleukin-1 beta in the rat by dietary N-3 fatty acid supplementation.. Prostaglandins Leukot Essent Fatty Acids.

[PDF_00483] Cooper A. L., Rothwell N. J. (1991). Mechanisms of early and late hypermetabolism and fever after localized tissue injury in rats.. Am J Physiol.

[PDF_00497] Endres S., Ghorbani R., Kelley V. E., Georgilis K., Lonnemann G., van der Meer J. W., Cannon J. G., Rogers T. S., Klempner M. S., Weber P. C. (1989). The effect of dietary supplementation with n-3 polyunsaturated fatty acids on the synthesis of interleukin-1 and tumor necrosis factor by mononuclear cells.. N Engl J Med.

[PDF_00480] Hardardottir I., Kinsella J. E. (1991). Tumor necrosis factor production by murine resident peritoneal macrophages is enhanced by dietary n-3 polyunsaturated fatty acids.. Biochim Biophys Acta.

[PDF_00503] Holt I., Cooper R. G., Hopkins S. J. (1991). Relationships between local inflammation, interleukin-6 concentration and the acute phase protein response in arthritis patients.. Eur J Clin Invest.

[PDF_00452] Kluger M. J. (1991). Fever: role of pyrogens and cryogens.. Physiol Rev.

[PDF_00455] Kunkel S. L., Chensue S. W., Phan S. H. (1986). Prostaglandins as endogenous mediators of interleukin 1 production.. J Immunol.

[PDF_00462] Lokesh B. R., Black J. M., German J. B., Kinsella J. E. (1988). Docosahexaenoic acid and other dietary polyunsaturated fatty acids suppress leukotriene synthesis by mouse peritoneal macrophages.. Lipids.

[PDF_00500] Lokesh B. R., Sayers T. J., Kinsella J. E. (1990). Interleukin-1 and tumor necrosis factor synthesis by mouse peritoneal macrophages is enhanced by dietary n-3 polyunsaturated fatty acids.. Immunol Lett.

[PDF_00459] Needleman P., Raz A., Minkes M. S., Ferrendelli J. A., Sprecher H. (1979). Triene prostaglandins: prostacyclin and thromboxane biosynthesis and unique biological properties.. Proc Natl Acad Sci U S A.

[PDF_00493] Philbrick D. J., Mahadevappa V. G., Ackman R. G., Holub B. J. (1987). Ingestion of fish oil or a derived n-3 fatty acid concentrate containing eicosapentaenoic acid (EPA) affects fatty acid compositions of individual phospholipids of rat brain, sciatic nerve and retina.. J Nutr.

[PDF_00468] Pomposelli J. J., Mascioli E. A., Bistrian B. R., Lopes S. M., Blackburn G. L. (1989). Attenuation of the febrile response in guinea pigs by fish oil enriched diets.. JPEN J Parenter Enteral Nutr.

[PDF_00457] Rola-Pleszczynski M., Lemaire I. (1985). Leukotrienes augment interleukin 1 production by human monocytes.. J Immunol.

[PDF_00453] Salmon J. A., Higgs G. A. (1987). Prostaglandins and leukotrienes as inflammatory mediators.. Br Med Bull.

[PDF_00488] Stock M. J. (1975). An automatic, closed-circuit oxygen consumption apparatus for small animals.. J Appl Physiol.

[PDF_00465] Terano T., Salmon J. A., Higgs G. A., Moncada S. (1986). Eicosapentaenoic acid as a modulator of inflammation. Effect on prostaglandin and leukotriene synthesis.. Biochem Pharmacol.

